# Sensory Processing and Attention Profiles Among Children With Sensory Processing Disorders and Autism Spectrum Disorders

**DOI:** 10.3389/fnint.2020.00022

**Published:** 2020-05-05

**Authors:** Jewel E. Crasta, Emily Salzinger, Mei-Heng Lin, William J. Gavin, Patricia L. Davies

**Affiliations:** ^1^Department of Occupational Therapy, Colorado State University, Fort Collins, CO, United States; ^2^Occupational Therapy Division, Ohio State University, Columbus, OH, United States; ^3^Fairfax County Public Schools, Fairfax, VA, United States; ^4^Center for Molecular and Behavioral Neuroscience, Rutgers University, Newark, NJ, United States; ^5^Department of Molecular, Cellular & Integrative Neuroscience, Colorado State University, Fort Collins, CO, United States; ^6^School of Music, Theatre, and Dance, Colorado State University, Fort Collins, CO, United States

**Keywords:** sensory processing disorders, autism, sensory processing, attention, children

## Abstract

This study explores the differences in the profile of relationships between sensory processing and attention abilities among children with sensory processing disorder (SPD), autism spectrum disorder (ASD), and typically developing (TD) children. The Test of Everyday Attention for Children (TEA-Ch), a performance-based measure of attention, was administered to 69 children (TD: *n* = 24; SPD: *n* = 21; ASD: *n* = 24), ages 6–10 years. All participants’ parents completed the Short Sensory Profile (SSP), a standardized parent-report measure of sensory-related behaviors. Discriminant analyses using the TEA-Ch and the SSP domains revealed two classification functions; the first revealed that both clinical groups significantly differed from the TD group with greater sensory processing challenges in the categories of auditory filtering, under-responsive/seeks sensation, low energy/weak, and taste/smell sensitivity subscales of the SSP. The second function discriminated between the two clinical groups, indicating that children with ASD had significantly greater control and sustained attention deficits and less sensory issues than did children with SPD. Together, the two functions correctly classified 76.8% of the participants as to their group membership. The different profiles of sensory processing and attention abilities in children with SPD and ASD may provide guidance in identifying appropriate individualized therapeutic strategies for these children.

## Introduction

Autism spectrum disorder (ASD) is a neurodevelopmental disorder characterized by persistent deficits in social communication and social interaction as well as restricted, repetitive patterns of behavior, interests, or activities (American Psychiatric Association, 2013). The prevalence rate of ASD is reported to be 1 in 69 children for children aged 8 years old (Christensen et al., 2018). The DSM-5, the diagnostic criteria for children with ASD, now includes deficits in sensory processing, namely, hyperreactivity or hyporeactivity to sensory input. However, another clinical condition that manifests with sensory issues is sensory processing disorder (SPD; Miller et al., 2007). As stated in the diagnostic manual for infancy and early childhood, SPD is diagnosed based on the presence of difficulties in detecting, modulating, interpreting, or organizing sensory stimuli to an extent that these deficits impair daily functioning and participation (Miller et al., 2005). Although children with SPD may have a comorbid diagnosis such as ASD, or attention deficit hyperactivity disorder (ADHD), SPD often occurs independently of recognized childhood psychopathologies (Goldsmith et al., 2006). The prevalence of sensory processing issues is reported to be around 1 in 20 to 1 in 6.25 children in the US general population (Ahn et al., 2004; Ben-Sasson et al., 2009), and a more recent study in Finland found the prevalence of sensory abnormalities to be around 8.3% in an epidemiological population of 8-year-old children (Jussila et al., 2020). Children with either SPD or ASD can have difficulties with processing sensation from tactile, auditory, visual, gustatory, olfactory, proprioceptive, and/or vestibular systems. Such children are often considered to have challenges in sensory integration (SI), which is the ability of the nervous system to process and organize sensory stimuli in the environment for adaptive functioning (Ayres’, 1972). These deficits can affect a child’s adaptive behavior, learning, coordinated movements, active playfulness, reading, and arithmetic abilities (Parham, 1998; Bundy et al., 2007). While children with either ASD or SPD may have deficits in sensory processing, their behavioral profiles of sensory processing may differ. A few studies have directly compared sensory processing characteristics in children with ASD and SPD (Schoen et al., 2009; Tavassoli et al., 2018). One study found lower physiological arousal and sensory reactivity in children with ASD than in those with SPD and higher reactivity after each sensory stimulus in the SPD group compared to the ASD group (Schoen et al., 2009). Although the neural substrates underlying sensory processing deficits in children with ASD and SPD remain to be elucidated, recent research has shown that larger gray matter volumes in early sensory regions are associated with atypical sensory processing of visual, auditory, tactile, and taste/smell modalities (Yoshimura et al., 2017). Neuroimaging studies have also found differences in white matter tracks between children with ASD and SPD (Chang et al., 2014), with abnormal posterior white matter microstructure correlating with sensory dysfunction in children with SPD (Owen et al., 2013). The current study sought to build on these studies to better differentiate the groups.

The focus of the current study was to understand the relationship of attention performance and successful sensory processing. Therapy using Ayres’ sensory integration theory (SIT) includes a specific focus on purposeful activities and requires an adaptive response and active participation by the child (Schaaf and Davies, 2010). Ayres’ (1972) SIT postulates that active *attention* is required for efficient sensory processing. Attention has been defined as the capacity to select various sensory input, perceptual objects, trains of thought, or courses of action for processing while other inputs, objects, thoughts, or actions are simultaneously occurring in a person’s environment (Talsma et al., 2010). Petersen and Posner (2012) described three distinct attention networks, each representing a different set of attentional processes, namely, selective, sustained, and attention control/shift.

Several researchers have shown that children with ASD have deficits in these three types of attention (Corbett et al., 2009; Christakou et al., 2013). In addition, deficits in joint attention, otherwise known as social attention, are considered a hallmark characteristic of the core manifestation in ASD (Zwaigenbaum et al., 2005). While social attention may be reduced in ASD, hyper-attention to, and abnormal exploration of, objects of circumscribed interests are also documented (Sasson et al., 2011). When considering attention performance in children with SPD, there is a paucity of research examining the specific types of attention deficits in children with SPD. Owen et al. (2013) found attention deficits, as measured by the inattention measure of the Sensory Profile, in 11 of the 16 children with SPD in their study, and Ahn et al. (2004) reported that around 40% of children with SPD show comorbid attention deficit symptoms. Children with SPD showed intermediate selective attention abilities on a visuomotor tracking task, with better performance than the ASD group but worse performance than typically developing (TD) controls (Brandes-Aitken et al., 2018). While there is some evidence supporting shared atypicality in the neural networks supporting attention and cognitive abilities in ASD and SPD (Owen et al., 2013), there may be different neural processes underlying attention deficits in these two distinct clinical conditions. In children with ASD, a lower degree of integration of information across cortical areas including frontal–parietal connectivity has been associated with attention and cognitive deficits (Just et al., 2007). However, there is limited research examining the neural basis of possible attention/cognitive deficits in children with SPD.

Difficulties in sensory processing and attention in children contribute to challenges in meaningful participation in everyday activities such as play (Leipold and Bundy, 2000; Bundy et al., 2007) and academic performance (DuPaul et al., 2001). Understanding the profile of both sensory processing and attention abilities in children with ASD and SPD will provide critical information that may distinguish children in these two clinical groups and guide interventions.

The purpose of the present study is to examine both sensory processing as measured by the Short Sensory Profile (SSP) and attention performance as measured by the Test of Everyday Attention for Children (TEA-Ch) among children with ASD and SPD and TD children. The study aimed not only to understand differences between groups but also explore the different groups’ profile of patterns of sensory processing and attention issues. We used discriminant analysis to identify the individual and combined contributions of specific sensory processing and attention abilities that would successfully predict the group membership of children into the respective groups, namely, ASD, SPD, and TD.

## Materials and Methods

### Participants

A total of 69 children aged 6–11 years (*M* = 7.83; *SD* = 1.26) participated in this study. The first group consisted of 24 children (19 males, five females; mean age 8.24 years, *SD* = 1.39) with a confirmed diagnosis of ASD (based on DSM-5) or Asperger’s syndrome/ASD (based on DSM-IV-TR) from a medical or psychological professional. Before recruitment into the study, the diagnosis of ASD was confirmed using the Asperger Syndrome Diagnostic Scale (ASDS; Myles et al., 2001), which was completed by the participant’s primary caregiver. At the inception of this study, the ASDS was one of the most valid assessments for diagnosing Asperger’s syndrome (Boggs et al., 2006). Based on parent report, the children with ASD did not have a comorbid diagnosis of ADHD or other neurodevelopmental disorders. The second group, 21 children with SPD (15 males, six females; mean age 7.54 years, *SD* = 1.42) were referred for this study by occupational therapists in the community who were treating the children for sensory processing issues. SPD group inclusion criteria were a community-based occupational therapy diagnosis of SPD plus a score in the definite difference range, defined as greater than two standard deviations from the mean, of either the total or auditory filtering score of the SSP (Chang et al., 2014). All children in the SPD group scored in the definite difference range on either the total or auditory filtering score on the SSP, except for one child who scored only one point less than the “definite difference” category range for the total score (i.e., 142). Based on parent report, the children with SPD did not have any other comorbid diagnoses such as ASD or ADHD. Third, a control group of 24 TD children (17 males, seven females; mean age 7.67 years, *SD* = 0.86) was composed of volunteers from the community who had no known physical, neurological, or behavior disorders and had not previously received any therapy services as reported by the parents. The TD children were age matched to the ASD group (*t*_(46)_ = −1.7, *p* = 0.1) and the SPD group (*t*_(43)_ = 0.4, *p* = 0.7). There was no age difference between the ASD and SPD groups (*t*_(43)_ = −1.67, *p* = 0.1).

All participants were part of a larger study that involved neuroimaging and behavioral tests, across two visits to the lab, with the SSP and TEA-Ch being collected on the first visit and Weschler’s Abbreviated Scale of Intelligence (WASI) collected on the second visit. Two children with ASD were not administered the WASI: one did not come for the second session, and the second child completed a neuroimaging portion on the second session but refused to do any of the behavioral tasks on the second visit. All children were verbal, and there was not a significant difference in IQ as measured by the two-scale WASI (Stano, 2004) between the three groups, *F*_(2, 61)_ = 2.170, *p* = 0.123; the mean (*SD*) IQ for each group was 112 (12.37) for TD, 109 (15.89) for SPD, and 103 (17.66) for ASD. *Post hoc* group comparisons using Scheffe confirmed that there were no significant group differences in IQ.

### Behavioral Measures

#### Short Sensory Profile (SSP)

The parent-report SSP was used to measure sensory behaviors, which is a standardized assessment tool frequently utilized to evaluate sensory processing in everyday activities. This tool is an abridged version of the Sensory Profile (Dunn, 1999). In the research sample of 1,037 children aged 3–10 years for the Sensory Profile, there was very little change in sensory processing abilities measured by the raw score across the age groups above 5 years (Dunn, 1999). Thus, the SSP raw scores above age 5 are relatively independent of age. The SSP has a reliability coefficient of 0.90, and discriminant validity is greater than 95% (McIntosh et al., 1999). The seven subscales assess auditory filtering, low energy/weak, under-responsive/seeks sensation, sensitivity to movement, tactile, taste/smell, and visual/auditory. Responses are scored on a 5-point Likert scale, with higher scores indicating better functional and adaptive behaviors. The SSP uses a classification system with cut off values to describe a child’s sensory processing abilities. A value in the “typical performance” classification indicates that the child performed better than the lowest 16% of the research sample (at or above the point of 1 *SD* below the mean). A value in the “probable difference” category indicates that the child performed like children in the lowest 14% of the research sample (scores at or above 2 *SD* below the mean but lower than 1 *SD* below the mean). A value in the “definite difference” classification indicates that the child performed like children in the lowest 2% of the research sample (below 2 *SD* below the mean). On the SSP, typical performance is indicated by a total score above 155, a probable difference is indicated by a total score ranging from 142 to 154, and a definite difference is indicated by a total score below 141. Parents of all the participants completed the SSP just prior to visiting the lab for the study.

#### Test of Everyday Attention for Children (TEA-Ch)

The TEA-Ch is a standardized (ages 6–16 years) and well-normed assessment that provides raw- and age-corrected standard scores for each of its nine subtests, namely, Sky Search, Score, Creature Counting, Map Mission, Score DT, Sky Search DT, Opposite Worlds, Walk Don’t Walk, and Code Transmission. The standard scores for each subtest range from 1 to 20, with 20 representing the best performance. The subtests combine to measure three attention subgroups, namely, selective, sustained, and attention control/shift, which correspond to Petersen and Posner (2012) attention networks (Manly et al., 1999). Manly et al. (2007) demonstrated that for 6- to 16-year-old Australian children, the age-standardized scores of the nine subtests can be combined into a three-factor configuration representing the three different types of attention, naming them as sustained, selective, and control/shift. In a more recent examination of the factor structure of the TEA-Ch conducted on children aged 6–13 years in the United States, the best-fitting model using structural equation modeling resulted in just two factors (Taylor et al., 2018). The first factor included the sustained subtests, and the second factor was a combination of the subtests representing both the selective and control/shift subtests. This structure of selective and control/shift collapsing into one factor is supported by Petersen and Posner (2012) who suggest that the neural networks for selective and control/shift may not be differentiated in young children. Taylor et al. (2018) named this combined factor control attention. Thus, the resulting two factors revealed in young children were sustained and control attention (Taylor et al., 2018). Based on Taylor et al. (2018), to obtain standardized coefficients, each participant’s standard score was multiplied by the unstandardized coefficients and then averaged to obtain the sustained and control attention domains (see Table 1 for unstandardized coefficient values). To categorize individuals into three attention performance categories, typical performance, probable difference, and definite difference, the mean and standard deviation (*SD*) of the total score (sustained + control) of the TD group were used as the standard score. Participants were classified in the typical performance category if their total scores were within 1 *SD* of the mean; in the probable difference category if their scores were between 1 *SD* below the mean and 2 *SD* below the mean; and in the definite difference category if scores were 2 *SD* below the mean. The TEA-Ch was administered to all participants at the lab. Participants who were unable to perform the practice trials provided in a given subtest received a score of 0 for that subtest.

**Table 1:** Unstandardized coefficients from the TEA-Ch model in Taylor et al. (2018), based on the full sample (*N* = 130) of that study, define two latent variables for attention, control and sustained.

Latent attention variable	TEA-Ch subtests	Unstandardized coefficient
**Control**	Sky Search	1.00
	Map Mission	0.716
	Opposite Worlds	1.675
	Creature Counting	0.652
**Sustained**	Code Transmission	1.00
	Walk Don’t Walk	0.760
	Score DT	0.783
	Sky Search DT	0.567

*To obtain an index of control and sustained attention for an individual using this model, the standard score for the four subtests within each attention domain (control and sustained) were multiplied by its associated unstandardized coefficient and then summed*.

### Data Analyses

For analysis of the SSP, the dependent measures included the raw scores of each subscale. Because raw scores are used for the SSP and ages varied some for the groups, correlations were conducted between age and the sensory profile scores to assure that the raw scores in the SSP were independent of age. Age did not correlate with any of the subscales or total sensory profile scores for any participant group, except for one subtest for the ASD group, taste/smell sensitivity, which was significantly associated with age (*r* = 0.48, *p* = 0.02). Age-standardized scores of the TEA-Ch were used to analyze group differences in attention. Consistent with the factor structure of the TEA-Ch found for young children by Taylor et al. (2018), the standard scores of the nine individual test items were consolidated to represent two subtypes of attention, sustained and control attention. Standard scores from the Sky Search (attention), Map Mission, Creature Counting (time), and Opposite Worlds (opposite) subtests were averaged to obtain a score representing control attention. Standard scores from Score, Code Transmission, Walk Don’t Walk, Score DT, and Sky Search DT were averaged to represent the sustained attention subtype. The TEA-Ch administration resulted in some missing data, which were determined as random after a review of the missing pattern graph and pattern frequencies. For 69 potential data points for each subtest, five of the TEA-Ch subtests had only three or fewer missing data points (Map Mission, Sky Search DT, Score, Score DT, and Walk Don’t Walk), one subtest (Sky Search—attention) had five missing data points (for these, the evaluator failed to enter the completion time, and the final score could not be calculated), one subtest (Code Transmission) had six missing data points, and finally Creature Counting (time) had 13 missing data points (due to a combination of the child not attempting the practice item, evaluator not recording the time, or the child not being able to count backward). Multiple imputation using procedures with the fully conditional specification Markov chain Monte Carlo method *via* the model type linear regression was conducted to provide estimates for the TEA-Ch missing values. The pooled estimates, an average of the values from five imputations, replaced the missing standard scores.

A multivariate analysis of variance (MANOVA) was used to examine group differences on the TEA-Ch sustained and control attention variables and SSP total score. *Post hoc* tests to examine group differences were performed using Tukey’s HSD. A total of three discriminant analysis procedures were used to evaluate group differences in the profiles of relationships across multiple sensory processing and attention variables. Discriminant analyses, a form of multiple regression, allow for the statistical determination of the significant importance of sensory processing and attention domains in classifying the groups. Justification for the sample size needed for discriminant analysis follows. Given the total number of participants in this study, the number of discriminating variables included in the discriminant analyses has been limited to an acceptable level. Related to discriminant analysis, Klecka et al. (1980) indicated that the total number of cases must exceed the number of variables by more than two. This study included nine discriminating variables, which would indicate the minimum number of participants would be 11. Discriminant analysis is analogous to multiple regression, except that in discriminant analysis, the dependent variable is nominal (Klecka et al., 1980). Related to multiple regression and a more conservative approach to determining the number of variables per number of participants, Brace et al. (2012) indicated that the number of participants must be five times the number of predictor variables. Using this conservative approach, this study with nine variables should have a minimum of 45 participants in total. Thus, the sample size of 69 is much greater than the minimum number necessary to conduct a valid analysis. The structure matrix represents correlations between variables in the model and examines how closely a variable is related to each function. The standardized canonical discriminant function coefficients represent the importance of each independent variable’s unique contribution to the discriminant function (McLachlan, 2004). All statistical analyses were performed using SPSS version 24.0 (IBM SPSS for Windows).

## Results

### Do Measures of Sensory Processing and Attention Differ Between Groups?

**Figure 1:**
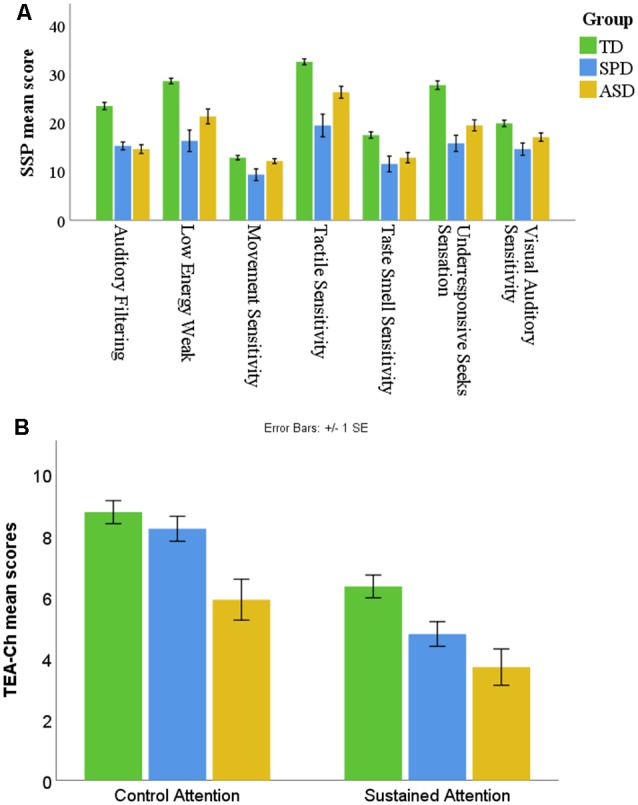
Comparisons of group differences for the two behavioral assessments: **(A)** the mean response of each group for each of the seven sensory domains of the Short Sensory Profile (SSP); **(B)** the mean response of each group for the attention subtypes derived from the Test of Everyday Attention for Children. The error bars represent 1 standard error of means (SE). Nonoverlapping error bars indicate the probable likelihood of significant differences between groups with a *p* < 0.05 if tested.

Table 2 displays the means and standard deviations for the seven subscales of the SSP for the three groups: TD children, children with SPD, and children with ASD. Children with SPD had the lowest means among the groups, followed by children with ASD, indicating that children with SPD had more sensory problems than children with ASD (see Figure 1A; Table 3 reports *F* statistics and *post hoc* tests to indicate differences between the groups on the total SSP score). Across all participants, age did not significantly correlate with the SSP domains (range of *r* values: 0.02–0.23, *p* > 0.05) or the TEA-Ch attention categories (range of *r* values: 0.02–0.19, *p* > 0.05). About 67% of the TD children had typical sensory performance, while 25% scored as probable difference and 8.3% (*n* = 2) scored as having a definite difference. For the SPD group, 90.5% scored as having a definite difference while two participants scored as having a probable difference. As expected, no SPD participants scored as having typical performance. For the ASD group, 70.8% scored as having definite difference and 25% as probable difference, and one participant scored as being in the typical performance category.

**Table 2:** Descriptive statistics and group differences on the Short Sensory Profile (SSP) and the Test of Everyday Attention for Children (TEA-Ch).

	Variables	TD children	SPD children	ASD children
SSP subscales (total raw scores)		Mean (SD)	Mean (SD)	Mean (SD)

	Auditory filtering	23.42 (3.7)	15.24 (3.7)	14.58 (4.45)
	Low energy	28.54 (2.84)	16.29 (10.1)	21.29 (7.44)
	Movement sensitivity	12.83 (2.22)	9.33 (5.48)	12.13 (2.49)
	Tactile sensitivity	32.50 (2.96)	19.43 (10.69)	25.25 (5.97)
	Taste/smell sensitivity	17.46 (3.19)	11.52 (7.46)	12.83 (5.19)
	Seeks sensation	27.71 (4.3)	15.76 (7.6)	19.46 (5.53)
	Visual/auditory	19.88 (3.19)	14.57 (5.8)	17.04 (4.23)
	Total	162.33 (15.2)	102.14 (41.56)	123.58 (24.26)
SSP percentiles	Typical	66.7% (*n* = 16)	0 (*n* = 0)	4.2% (*n* = 1)
	Probable difference	25% (*n* = 6)	9.5% (*n* = 2)	25% (*n* = 6)
	Definite difference	8.3% (*n* = 2)	90.5% (*n* = 19)	70.8% (*n* = 17)
TEA-Ch domains (standard scores)	Sustained attention	6.33 (1.83)	4.78 (1.85)	3.7 (2.9)
	Control attention	8.77 (1.84)	8.22 (1.87)	5.91 (3.28)
TEA-Ch percentiles	Typical	79.2% (*n* = 19)	71.4% (*n* = 15)	41.7% (*n* = 10)
	Probable difference	16.6% (*n* = 4)	23.8% (*n* = 5)	20.8% (*n* = 5)
	Definite difference	4.2% (*n* = 1)	4.8% (*n* = 1)	37.5% (*n* = 9)

*Note: TD, typically developing; SPD, sensory processing disorder; ASD, autism spectrum disorder*.

**Table 3:** MANOVA statistics and *post hoc* Tukey’s HSD depicting group differences on the Short Sensory Profile (SSP) and the Test of Everyday Attention for Children (TEA-Ch).

MANOVA variables	Between-subject effects *F*, *p*	TD vs. SPD Mean difference, *p*	TD vs. ASD Mean difference, *p*	SPD vs. ASD Mean difference, *p*
SSP total	26.18, *p* < 0.0005	60.19, *p* < 0.0005	38.75, *p* < 0.0005	−21.44, *p* = 0.037
Control attention	9.16, *p* < 0.0005	0.54, *p* = 0.74	2.86, *p* < 0.0005	2.32, *p* = 0.006
Sustained attention	8.18, *p* = 0.001	1.55, *p* = 0.06	2.63, *p* < 0.0005	1.08, *p* = 0.26

*Note: TD, typically developing children; SPD, sensory processing disorder; ASD, autism spectrum disorder*.

Table 2 also displays the means and standard deviations for the two subtypes of attention (sustained and control) as measured by the TEA-Ch for the three groups: TD children, children with SPD, and children with ASD (Table 3 reports *F* statistics and *post hoc* tests to indicate differences between the groups on each subtype). The means indicate that children with ASD had significantly greater attention issues (lower scores) on control and sustained attention compared to TD peers and on control attention compared to children with SPD. Children with SPD did not significantly differ from TD peers on control attention, and the difference on sustained attention trended towards significance (*p* = 0.06; see Figure 1B). About 79% of the TD children had typical attention abilities, and 16.7% scored as probable difference, while only one participant scored as having a definite difference. For the SPD group, 71.4% scored as having typical performance, while 23.8% scored as having a probable difference and only one participant (4.8%) scored as having a definite difference. Interestingly, for the ASD group, 41.7% scored as having a definite attention issue, 20.8% scored as having a probable difference, and only 37.5% scored as having typical performance (see Table 2).

### Do Measures of Sensory Processing Alone Predict Group Membership?

The first discriminant analysis evaluated how well the seven subscales of the SSP alone could correctly classify each child’s group membership. First, two functions were obtained when predicting membership for groups. Function 1 significantly separated the TD group from the ASD and SPD groups (*λ* = 0.27, *p* < 0.0005); however, the second function separating the ASD and SPD groups was not significant (*λ* = 0.83, *p* = 0.07). Secondly, these two functions correctly classified 72.5% of all participants compared to their group membership. The TD group had 95.8% correct classification, while 58.3% of children with ASD were correctly classified and 61.9% of children with SPD were correctly classified.

### Do Attention Abilities Alone Predict Group Membership?

The second discriminant analysis evaluated how well the two attention domains alone could correctly classify each child’s group membership. Function 1 significantly separated the TD group from the ASD and SPD groups (*λ* = 0.72, *p* < 0.0005); however, the second function separating the ASD and SPD groups was not significant (*λ* = 0.95, *p* = 0.07). The function correctly classified 52.2% of all participants compared to their group membership. The TD group had 58.3% correct classification, while 54.2% of children with ASD were correctly classified and 42.9% of children with SPD were correctly classified.

### Does the Combination of Sensory Processing and Attention Abilities Predict Group Membership?

**Figure 2:**
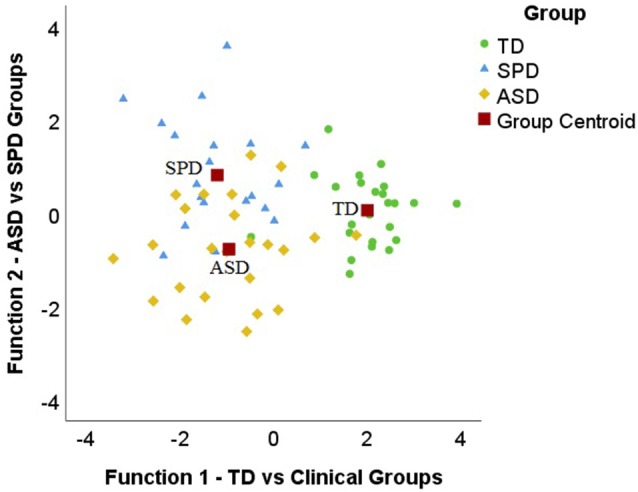
Territorial map for the full discriminant analysis model. The small circles, triangles, and diamonds represent individuals of their respective groups plotted according to the two functions. The *x*-axis represents function 1, which significantly separates the typically developing (TD) group from the two clinical groups of autism spectrum disorder (ASD) and SPD. The *y*-axis represents function 2, which significantly separates the ASD and SPD children. The red squares represent the centroid (i.e., group means).

A third discriminant analysis using the scores from the SSP subscales and the TEA-Ch domains examined the unique contribution of sensory processing and attention in discriminating between the three groups (see Table 4). Function 1 significantly separated the TD group from the two clinical groups of ASD and SPD (*λ* = 0.22, *p* < 0.0005). Additionally, function 2 significantly separated the ASD and SPD children (*λ* = 0.70, *p* = 0.005; see Figure 2). Based on the canonical structure matrix factor loadings (see Table 4), the variables in function 1 that significantly discriminated the TD group from the two clinical groups were the auditory filtering, under-responsive/seeks sensation, low energy/weak, and taste/smell sensitivity subscales of the SSP. Thus, the two clinical groups were most different from the TD group in their scores on these four variables. Similarly, the variables in function 2 that significantly discriminated the two clinical groups were tactile sensitivity, movement sensitivity, and visual/auditory sensitivity subscales of the SSP, along with the control and sustained attention domains of the TEA-Ch. Interestingly, of the variables that loaded on function 2, children with SPD had more sensory issues (lower means) for the three subscales of the SSP compared to the ASD group. This indicates that the SPD group had more sensory processing issues in tactile sensitivity, movement sensitivity, and visual/auditory sensitivity compared to children with ASD. However, children with ASD had greater deficits (lower means) in the control and sustained attention domains than the SPD group. This suggests that the ASD group has more attention deficits compared to the SPD group. These two functions correctly classified 76.8% of the participants to their group membership. The TD group had 95.8% correct classification, while 66.7% of children with ASD were correctly classified and 66.7% of children with SPD were correctly classified. Thus, the combination and profile of attention and sensory processing characteristics led to better discrimination between the three groups, TD children, children with ASD, and children with SPD, than either sensory processing or attention characteristics alone.

**Table 4:** The results of the discriminant analysis that included measures from the Short Sensory Profile and the Test of Everyday Attention for Children to predict each participant’s group membership.

Variables	Structure matrix		Standardized canonical	
	Function 1	Function 2	Function 1	Function 2
Auditory filtering	0.691*	0.213	0.740	0.264
Under-responsive/seeks sensation	0.560*	−0.308	0.383	−0.136
Low energy/weak	0.439*	−0.364	0.522	−0.065
Taste/smell sensitivity	0.315*	−0.101	−0.329	0.537
Control attention	0.217	0.631*	−0.076	−0.712
Tactile sensitivity	0.449	−0.536*	0.725	−0.907
Movement sensitivity	0.195	−0.458*	−0.397	0.082
Visual auditory sensitivity	0.295	−0.347*	−0.633	−0.072
Sustained attention	0.299	0.301*	0.248	−0.037

Pooled within-groups correlations between discriminating variables and standardized canonical discriminant functions reported in the structure matrix. *Largest absolute correlation between each variable and any discriminant function; variables ordered by the absolute size of correlation within a function within the structure matrix.

## Discussion

The purpose of this study was to determine if the profiles of sensory processing and attention abilities in children with SPD and children with ASD differed in a systematic manner. As expected, the group means of the SSP and the TEA-Ch measures indicated that children with SPD and ASD had more sensory processing issues and attention deficits as compared to TD children. For the SPD group, all participants scored as having either a probable or definite sensory processing deficit, while only 28.6% scored as having either a probable or definite attention deficit. For the ASD group, 95.8% scored as having either a probable or definite sensory processing deficit, while 58.3% scored as having either a probable or definite attention deficit. As expected in the general population, 8.3% of the TD group (*n* = 2) scored as having a definite sensory deficit (Ahn et al., 2004; Jussila et al., 2020), and one participant (4.2%) scored as having a definite attention deficit. The discriminant analyses also indicated that the profile of sensory processing and attention challenges differ in the SPD and ASD groups such that children with SPD had more sensory processing issues than the ASD group, while the ASD group had more attention deficits than the SPD group, especially in control attention. Thus, the profile of attention and sensory processing issues significantly differentiate children with ASD and SPD. This study used a novel approach by concurrently using sensory processing and attention abilities to understand differences in functional performance in children with ASD and children with SPD. The findings of this study can help clinicians and therapists identify specific therapeutic strategies tailored to the child’s diagnosis-specific profile of strengths and weaknesses.

### Sensory Processing in Children With SPD and in Children With ASD

Researchers have found that children with SPD have differences in both behavioral and neurophysiological measures of sensory processing as compared to TD peers. Owen et al. (2013) found that the degree of abnormal posterior white matter microstructure correlated with sensory behavior as measured by the Sensory Profile. Using electroencephalography (EEG) measures obtained from the sensory gating paradigm, Davies and Gavin (2007) demonstrated differences in filtering auditory information between children with SPD and their neurotypical peers. Their results showed that children with SPD had significant difficulties in filtering out repeated auditory input and lacked the ability to selectivity regulate their sensitivity to sensory information (Davies and Gavin, 2007; Davies et al., 2009).

In addition, the results of this current study indicated that, in general, children with SPD may have more sensory processing issues than children with ASD (see Table 2). These results are partially supported by a study examining physiological and behavioral differences in sensory processing between children with ASD and children with sensory modulation disorder (SMD; Schoen et al., 2009). SMD is a subtype of SPD and refers to an extreme inability to regulate responses to everyday sensory information to which most people in the general population easily acclimate (James et al., 2011). Both the clinical groups (ASD and SMD) showed greater sensory issues compared to the TD controls (Schoen et al., 2009). Schoen et al. (2009) found that the ASD group had greater issues in the tactile sensitivity, low energy/weak, and taste/smell sensitivity subscales. Contrary to the Schoen et al. (2009) study, the sample of children with SPD in the current study had greater sensory processing issues, including more sensory issues in the tactile sensitivity subscale of the SSP, compared to the ASD group. Further research is required to explicate the differences in the sensory profiles of children across the autism spectrum and across the SPD subcategories. In the current study, 95.8% of the participants with ASD scored as having either a probable or definite difference in sensory processing. This finding is consistent with literature stating that 42% to 95% of the children with autism exhibit sensory processing issues as measured by the Sensory Profile, a parent-report measure (Liss et al., 2006; Tomchek and Dunn, 2007).

Researchers have identified sensory subtypes in ASD in an effort to reduce the heterogeneity of clinical features, with Ausderau et al. (2014) and Lane et al. (2014) identifying several subtypes of which two subtypes reflect the severity of sensory issues; for one subtype, members exhibit fewer sensory issues, while for the second subtype, members display the most sensory issues. These two research groups also identified several other subtypes that specify particular responses to sensory input (i.e., “sensitive-distressed” and “attenuated-preoccupied, ” Ausderau et al., 2014; and “taste/smell sensitivity” and “postural inattentive, ” Lane et al., 2014). The differences in the subtypes identified by these two research groups may be attributed to the differences in sensory modalities examined and assessment tools used in the respective research. In children with SMD, two sensory subtypes have been identified, the first is characterized by sensory seeking/craving, hyperactive, unsocial, and impaired cognitive/social behavior, while the second is characterized by movement sensitivity, emotional withdrawal, and low energy/weak (James et al., 2011). Further research is required to understand the complexities of sensory subtypes and implications for clinical practice for both ASD and SPD/SMD.

In the current study, 8.3% of the TD group scored as having a definite difference in sensory processing. This is also consistent with literature stating that sensory processing challenges are present in 5–13% of the general population of young children (Ahn et al., 2004; Ben-Sasson et al., 2009; Jussila et al., 2020).

### Attention Abilities in Children With SPD and in Children With ASD

Petersen and Posner (2012) described the subtypes of attention as involving distinct brain networks, which interact with each other to enable an individual to perform the complex tasks of everyday life. The findings of the current study indicate that children with ASD have deficits in sustained and control attention compared to a group of TD children. Impairments in orienting (Zwaigenbaum et al., 2005), sustained attention (Garretson et al., 1990), vigilance and cognitive flexibility/switching (Corbett et al., 2009), and shifting and disengaging attention (Hill, 2004; Elsabbagh et al., 2009) have been consistently reported in individuals with ASD. Researchers have posited that early deficits in disengaging attention in infants with ASD may lead to the cascade of ASD symptomatology and the emergence of the broader phenotype including sensory deficits (Elsabbagh et al., 2009; Franchini et al., 2019). Although the current study did not find significant differences in control or sustained attention between children with SPD and TD children, the group difference on sustained attention trended towards significance, suggesting that, in general, children with SPD may present with deficits in sustained attention. Additionally, the means on the TEA-Ch subtypes (see Figure 1B) indicate that children with SPD had greater attention issues than their typical peers, suggesting that attention may be an important cognitive domain that may be incorporated during therapy. A recent study found similar results when comparing cognitive control in children with ASD, SPD, and TD controls using visuomotor tracking and tracing skills wherein the ASD group had greater deficits than the SPD and TD groups and that the SPD group had intermediate abilities—performing above the ASD group but below the TD group (Brandes-Aitken et al., 2018). In the diagnostic manual for infancy and early childhood, Miller et al. (2005) stated that deficits in attention are commonly found in children with regulatory-sensory processing disorders (RSPD). They proposed that deficits in attention observed in children with RSPD may stem from poorly organized or modulated sensory processing. A study examining differences in behavior of children with ADHD and children with SMD compared to neurotypical peers found that children with SMD and ADHD had more attentional problems than the TD group (Miller et al., 2012). The study conducted by Miller et al. (2012) used parent-report measures, Leiter-P parent report, and the Child Behavior Checklist, to obtain an attention score, whereas the current study used a performance-based measure of attention. To our knowledge, this is the first study to examine attention abilities using a performance-based assessment in children with SPD. Further research in a larger sample size is required to confirm and expand the findings of this study regarding attention in children with SPD.

The differences in the pattern of sensory processing and attention abilities in children with ASD and children with SPD highlight the distinctness of the two clinical conditions. Demopoulos et al. (2017) examined auditory and somatosensory cortical processing using magnetoencephalographic (MEG) data and showed that children with ASD had greater auditory processing deficits than SPD and TD peers, while somatosensory processing was similar between ASD and SPD groups. These differences highlight the importance of understanding the difference between attention and sensory processing patterns between children with ASD and SPD using both behavioral and neuroimaging methods. While both ASD and SPD show decreased white matter connectivity in areas associated with sensory processing and cognitive control (Owen et al., 2013), there may be greater involvement of neural structures underlying attention and cognitive control in children with ASD compared to children with SPD.

The small sample size of the current study limits the generalizability of the study findings. This study used the SSP to measure sensory processing since this is the most widely used tool in research studies; however, future research should include observation-based measures of sensory processing. One subtest of the TEA-Ch had 13 missing data points; however, we used a multiple-imputations procedure to minimize the effects of missing data. The current study used the Asperger Syndrome Diagnostic Tool for confirmation of ASD diagnosis; however, because there are overlapping comorbidities in these clinical populations, more robust tools such as the Autism Diagnostic Observation Schedule, second edition (ADOS-2), and the Social Responsiveness Scale, second edition (SRS-2), should be used in further studies. Further research with larger sample sizes should examine the relationship between age and sensory and attention functions.

## Conclusion

The study findings indicated that children with ASD and children with SPD have different sensory processing and attention profiles. Specifically, children with SPD tend to have more sensory processing issues than children with ASD, whereas children with ASD tend to have more attention deficits than children with SPD. Compared to TD children, the ASD group had challenges in both subtypes of attention, namely, sustained and control attention (Figure 1B), while the SPD group appeared to have some difficulty in sustained attention. Also, children with ASD have more deficits in control attention than the SPD group (Figure 1B). These results can help therapists identify specific treatment strategies while working on attention and sensory processing in children with SPD and ASD. The results of this study indicate that the profiles of abilities and challenges are unique for the ASD and SPD groups. These findings suggest that for children with SPD, therapy should emphasize sensory-based strategies while including global attention tasks. Whereas for children with ASD, therapy should prominently consider global attention training along with sensory-based techniques.

## Data Availability Statement

The data will be made available to interested researchers. To access the data, researchers should directly contact the corresponding author (patricia.davies@colostate.edu).

## Ethics Statement

Upon visiting the lab, parents provided informed consent and the child participants provided assent. The Colorado State University’s institutional review board reviewed and approved all procedures used in this study.

## Author Contributions

JC: data collection, data entry, statistical analysis, funding acquisition, writing—original draft. ES: data collection, data entry, and writing—original draft. M-HL: data collection, data entry, and statistical analysis, writing—review, and editing. WG: study conceptualization, supervision, resources, funding acquisition, writing—review, and editing. PD: study conceptualization, data collection, statistical analysis, supervision, resources, funding acquisition, writing—review, and editing.

## Conflict of Interest

The authors declare that the research was conducted in the absence of any commercial or financial relationships that could be construed as a potential conflict of interest.
